# The Joint Mobile Emerging Disease Clinical Capability (JMEDICC) laboratory approach: Capabilities for high-consequence pathogen clinical research

**DOI:** 10.1371/journal.pntd.0007787

**Published:** 2019-12-19

**Authors:** Prossy Naluyima, Willy Kayondo, Chi Ritchie, Joseph Wandege, Sharon Kagabane, Lydia Tumubeere, Brenda Kusiima, Daniel Kibombo, Sharon Atukunda, Christine Nanteza, Harriet Nabirye, Francis Bunjo Mugabi, Sarah Namuyanja, Christopher Hatcher, Hypaitia Rauch, Moses Mukembo, Patrick Musinguzi, JMEDICC Consortium, Nathan Sanders, Elizabeth Turesson, Christian Cando, Richard Walwema, Derrick Mimbe, Janice Hepburn, Danielle Clark, Mohammed Lamorde, Hannah Kibuuka, Saima Zaman, Anthony P. Cardile, Karen A. Martins

**Affiliations:** 1 Makerere University Walter Reed Project, Kampala, Uganda; 2 US Army Medical Research Institute of Infectious Diseases, Fort Detrick, Maryland, United States of America; 3 Infectious Diseases Institute, College of Health Sciences, Makerere University, Kampala, Uganda; 4 Fort Portal Regional Referral Hospital, Fort Portal, Uganda; 5 The Tauri Group, Alexandria, Virginia, United States of America; 6 Pharmacopeia, Rockville, Maryland, United States of America; 7 Biological Threat Reduction Program, Fort Belvoir, Virginia, United States of America; 8 Henry M. Jackson Foundation, Bethesda, Maryland, United States of America; University of Geneva Hospitals, SWITZERLAND

## Abstract

Following the 2013–2016 Ebola virus outbreak in West Africa, numerous groups advocated for the importance of executing clinical trials in outbreak settings. The difficulties associated with obtaining reliable data to support regulatory approval of investigational vaccines and therapeutics during that outbreak were a disappointment on a research and product development level, as well as on a humanitarian level. In response to lessons learned from the outbreak, the United States Department of Defense established a multi-institute project called the Joint Mobile Emerging Disease Intervention Clinical Capability (JMEDICC). JMEDICC’s primary objective is to establish the technical capability in western Uganda to execute clinical trials during outbreaks of high-consequence pathogens such as the Ebola virus. A critical component of clinical trial execution is the establishment of laboratory operations. Technical, logistical, and political challenges complicate laboratory operations, and these challenges have been mitigated by JMEDICC to enable readiness for laboratory outbreak response operations.

## Author summary

Conducting clinical research during high-consequence pathogen outbreaks engenders complications not experienced in standard clinical research. In addition to regulatory expertise, international stakeholder engagement—including community outreach into research-naive local communities—is required, as are the technical clinical, laboratory, and infection prevention and control expertise that enables the safe and effective execution of the study in hazardous circumstances. Since the 2013–2016 Ebola virus outbreak in West Africa, researchers and public health officials alike have recognized the need to discuss these challenges. The Joint Mobile Emerging Disease Intervention Clinical Capability (JMEDICC) is a unique project tasked with pre-positioning a team of highly skilled clinical researchers to execute therapeutics clinical trials during a filovirus outbreak. The project has been operating in Fort Portal, Uganda, since 2016 and is preparing to conduct clinical research if the current Ebola outbreak in the Democratic Republic of Congo spreads to Uganda. The capability is not necessarily specific for filoviruses, however, but could be utilized for high-containment clinical trial work for other high-consequence pathogens.

## Introduction

The 2013–2016 Ebola virus outbreak in West Africa was the largest filovirus outbreak in recorded history, with over 28,000 suspected, probable, and confirmed cases and over 11,000 deaths across 10 nations [1]. The causes of the breadth and intensity of this outbreak have been rigorously analyzed in an effort to prevent such a humanitarian catastrophe from occurring again [2–6]. Several factors impacted the spread of the virus, including cultural beliefs, (most specifically, burial practices), insufficient training of healthcare workers (HCWs), and lack of community awareness about the causes of viral hemorrhagic fever (VHF). An Ebola virus outbreak had never before been reported in Liberia, Sierra Leone, and Guinea, delaying recognition of the outbreak and complicating community education and sensitization. Moreover, the virus killed a striking number of HCWs, devastating the public health infrastructure in the region with consequences that persist to this day in some areas [7, 8].

In August of 2014, World Health Organization (WHO) announced that the outbreak met conditions of a Public Health Emergency of International Concern and encouraged international response teams to increase aid [9]. Despite the capabilities with which these teams arrived, they were faced with a number of challenges, including delays in obtaining diagnostic test results, unstable national power grids, adulterated fuel, limited internet access, and staff turnover due to factors including fear, infection, and lack of pay. Moreover, the lack of any approved therapeutic or vaccine with which to treat impacted populations limited the capabilities of even the most skilled teams, frustrating attempts to gain community trust and prevent further spread of the virus.

Toward the end of the 2013–2016 outbreak, response entities recognized that efforts focused solely on patient care tragically missed an opportunity to determine whether specific medical countermeasures (MCMs) were safe and efficacious and could potentially prevent or halt future outbreaks [10–15]. The rVSV-ZEBOV (Merck) vaccine was tested in a ring vaccination trial in Guinea, with potentially promising results [16]. In addition, the therapeutics TKM-130803 (Tekmira Pharmaceuticals) and favipiravir (Toyama Chemical) were tested in “single-arm” clinical trials: favipiravir was tested in Guinea in the single-arm JIKI trial [17, 18] and TKM-130803 was tested in Sierra Leone [19]. ZMapp (Mapp Biopharmaceutical), an antibody cocktail, was the only drug tested in a multi-arm clinical trial with standard of care as the control arm [20]. The trial enrolled in Liberia, Sierra Leone, Guinea, and the US, but, unfortunately, was initiated in the waning months of the outbreak, preventing collection of adequate data to determine efficacy.

At the writing of this manuscript, a new Ebola virus outbreak persists in the Democratic Republic of the Congo (DRC). The outbreak is located in the far eastern part of the country and began in North Kivu, a region besieged by civil conflict. Ongoing conflict among local insurgent groups has significantly impeded the ability of international groups to respond to the outbreak and has put HCWs and response entities at risk. Despite the untenable conditions, advances in clinical research capabilities are being made. Expanded access protocols adhering to the Monitored Emergency Use of Unregistered and Investigational Interventions (MEURI) framework that has been promoted by WHO have been initiated for several investigational drugs, and the rVSV-ZEBOV vaccine is currently being administered in the DRC, South Sudan, Rwanda, and Uganda. In addition, a four-arm clinical trial was recently concluded in the DRC, comparing the efficacy of ZMapp and REGN-EB3 (Regeneron Pharmaceuticals), both antibody cocktails; mAb114 (Ridgeback Biotherapeutics LP), a monoclonal antibody [21]; and remdesivir (Gilead Sciences, Inc.), an antiviral small molecule [22]. On August 12, 2019, authorities at the US National Institutes of Health and Institut National de Recherche Biomédicale (INRB) announced the preliminary results of the study, finding that 2 of the 4 products were effective at reducing mortality of Ebola virus–infected patients [23]. Execution of this trial was a monumental challenge for investigators considering the civil unrest in the region and other barriers to research in an outbreak setting. In this article, we will describe the hurdles and solutions identified by JMEDICC in fielding this kind of research, focusing on laboratory-specific challenges and solutions. The JMEDICC approach is by no means the only approach to clinical research in an outbreak setting; we hope that this article will lead to discussion that directs optimization of procedures and may be used by other groups to facilitate their outbreak response plans and policies.

## Project overview

JMEDICC was established in 2015 in answer to many of the lessons learned from the West Africa Ebola outbreak. The project is a proactive effort to establish clinical research capacity in an area at risk of filovirus outbreaks. The primary goal is to obtain clinical data that will support US Food and Drug Administration (FDA) licensure of a therapeutic effective against filoviruses or other high-consequence pathogens [24–26]. JMEDICC, currently operational in western Uganda, is a collaborative effort among US and Ugandan partners, including the Henry M. Jackson Foundation for the Advancement of Military Medicine (HJF), US Army Medical Research Institute of Infectious Diseases (USAMRIID), the Naval Medical Research Center (NMRC), the Makerere University Walter Reed Project (MUWRP), the Infectious Diseases Institute (IDI), and the funding organization, Joint Project Manager, Chemical, Biological, Radiological, and Nuclear Medical (JPM CBRN Medical), Joint Program Executive Office for Chemical, Biological, Radiological and Nuclear Defense, US Department of Defense. In addition to the primary partners, the project is supported by outside consultants from institutes including Boston University and Walter Reed Army Institute of Research, as well as contract organizations that assist with logistics and regulatory path.

**Fig 1:**
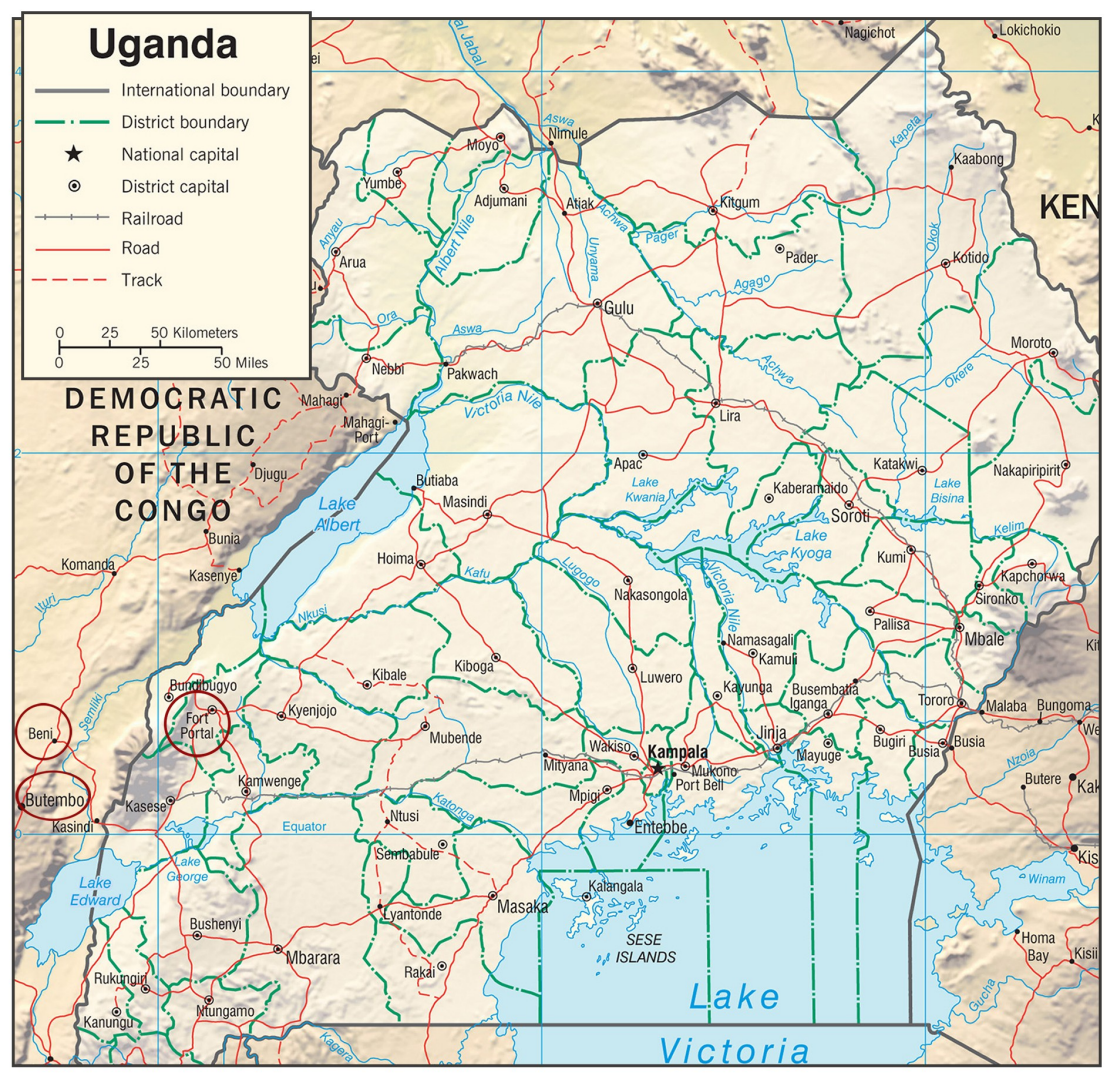
Map of Uganda and the bordering area of the DRC. Fort Portal, the JMEDICC hub site in Uganda, is located in the western part of Uganda near the border with the DRC and is circled in red; Beni and Butembo in DRC, sites of the 2018–present DRC Ebola virus outbreak, also shown and circled in red. *Map taken from the CIA website the Uganda Physiography map* https://www.cia.gov/library/publications/resources/cia-maps-publications/Uganda.html. CIA, Central Intelligence Agency; DRC, Democratic Republic of Congo; JMEDICC, Joint Mobile Emerging Disease Intervention Clinical Capability.

In western Uganda, the JMEDICC effort is based out of the Fort Portal Regional Referral Hospital (FPRRH), where the hospital director, hospital administrator, district health office, and other Ministry of Health (MoH) staff have been crucial to the integration of the project into hospital practices and procedures. FPRRH was selected as the hub site because of its proximity to previous filovirus outbreak sites, including Bundibugyo and Kasese (Fig 1) [27]. The hospital offered the project a research ward that JMEDICC renovated to provide clinical capacity for 6 severely ill patients at a time; the unit also has a separate laboratory building and a storage area, which ensure buffer stocks of critical materials.

A full-time team of 22 clinical, laboratory, and logistics personnel is located at FPRRH, with support from US-based subject matter experts and consultants from MUWRP and IDI in Kampala. The Fort Portal staff is engaged in the ongoing execution of an observational sepsis protocol coordinated by the Austere environments Consortium for Enhanced Sepsis Outcomes (ACESO). The study provides staff with full-time employment that hones their clinical research, triage, and acute patient care expertise, and it generates inherently valuable research data as an independent study. Concurrent with study execution, the team receives training in infection prevention and control (IPC), biosafety, and outbreak response. The project vision is that, in the event of an outbreak, the training that the team has received through execution of the observational sepsis study will enable focus on patient care, collection of high-quality data, and their own personal safety, minimizing the number of new research-oriented processes and procedures that must be learned during the outbreak response.

In addition to development of the technical capability to conduct a clinical trial, the team is actively engaged in the advancement of clinical trial protocols. An aim of JMEDICC is to establish pre-positioned clinical trial protocols in Uganda for Investigational New Drug (IND) therapeutics that might be activated in the event of an outbreak. This effort includes regular engagement with Ugandan Institutional Review Boards (IRBs), the National Drug Authority (NDA), the Uganda National Council for Science and Technology (UNCST), US IRBs, and the Department of Defense Human Research Protection Office.

**Fig 2:**
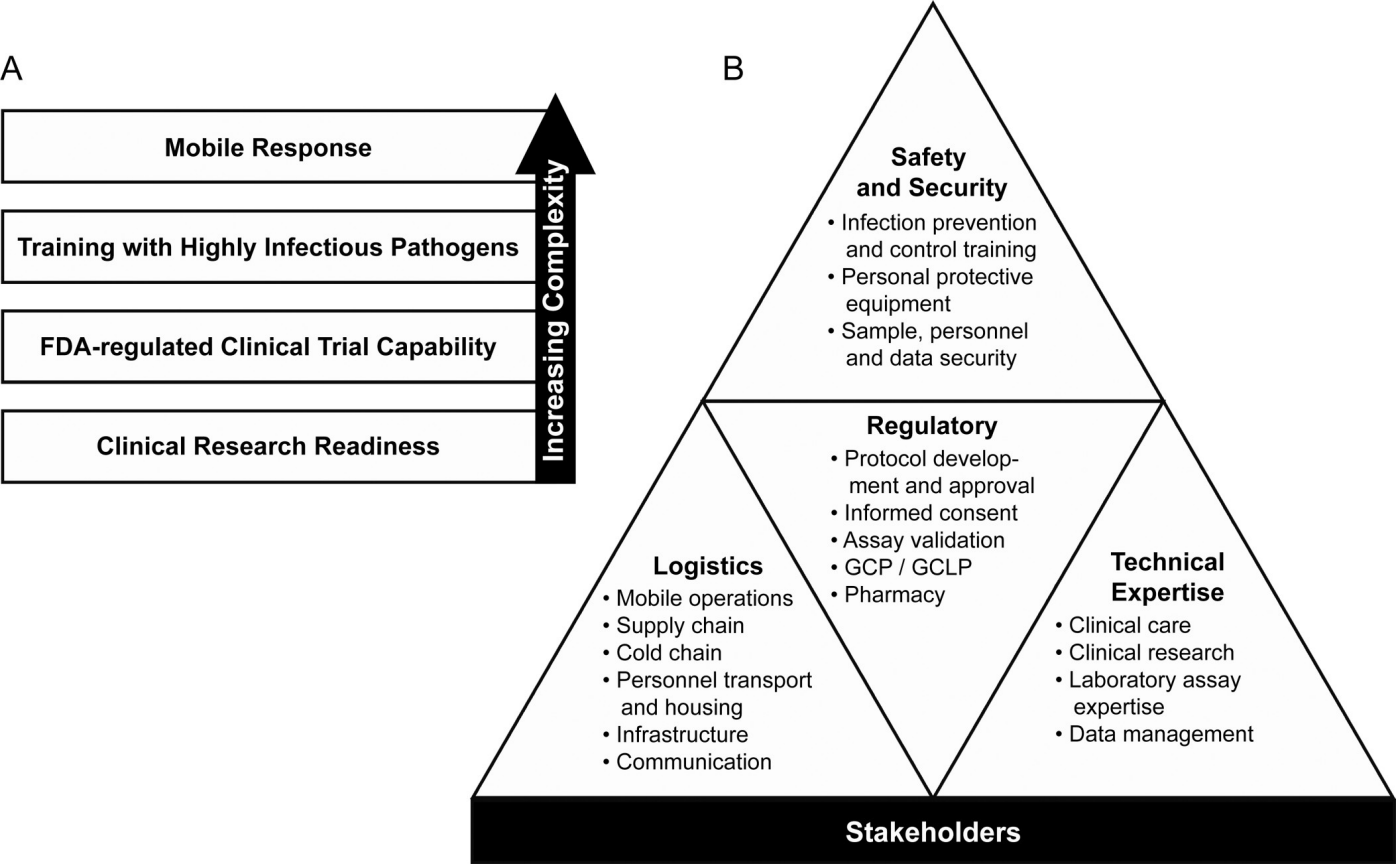
JMEDICC execution schematic highlighting (A) JMEDICC approach of increasing complexity as competency is established and (B) the multifaceted nature of the project preparedness and response. JMEDICC, Joint Mobile Emerging Disease Clinical Capability.

The JMEDICC strategy for outbreak preparedness is to increase the complexity of drills and training as the team’s capability improves (Fig 2A). Establishing high-quality, auditable, clinical research capabilities is an essential first step, including good clinical practice (GCP), good clinical laboratory practice (GCLP), appropriate instrumentation, and data management systems that are compliant with international standards. Next is to develop the necessary biosafety, decontamination, and biosecurity measures necessary to work in a filovirus or other high-consequence pathogen outbreak setting. This includes adapting data management and laboratory assays to a limited-access isolation environment [28–30]. Finally, the ultimate goal is to enable a mobile response capable of relocating to an outbreak outside the immediate vicinity of FPRRH. Mobility would permit flexibility in response scope. The JMEDICC end product is built upon stakeholder engagement and support, strong logistics and technical expertise, established clinical protocols and standard operating procedures meeting regulatory requirements, and biosafety and security for personnel, products, and data (Fig 2B). Sustaining this capability requires long-term commitment and investment.

## Personnel readiness

Competent and prepared personnel are critical to the effective deployment of a clinical trial during a disease outbreak. The US-based JMEDICC team chose to partner with local research organizations MUWRP and IDI to execute the project rather than rely on a traveling team of external subject matter experts (SMEs) that would be activated during an outbreak event. MUWRP and IDI have extensive experience conducting clinical research of infectious diseases and thus had a framework suitable to be adapted to JMEDICC activities. This framework included existing partnerships with the MoH, integration within the Ugandan national outbreak response mechanism, experience in and processes for navigating Ugandan regulatory requirements, and regular community outreach and education activities. The model of empowering a local national team for project execution is pragmatic in terms of minimizing outbreak response time and increasing team integration with local outbreak response partners; critically, it also builds a local capability with expertise in IPC and outbreak response that can propagate within Uganda through train-the-trainer programs and partnership.

At the FPRRH laboratory, JMEDICC staff work alongside hospital staff. This arrangement has depended on durable and respectful lines of communication that enable maintenance of JMEDICC’s scope of work while ensuring a harmonious working environment. The JMEDICC project recruited 5 full-time laboratory staff, a team that was recently expanded to 8 because of additional project requirements. Staff members’ work is heavily coordinated with the hospital’s laboratory team, with project roles including an onsite laboratory lead, a lead for laboratory logistics, the IPC and biosafety leads, and a molecular biology lead. In addition, the quality support team works closely with the FPRRH laboratory quality officer to support and strengthen the Quality Assurance and Quality Control (QA/QC) section of the laboratory and ensure compliance with standards, as per ISO15189.

## Laboratory quality management

During the West Africa outbreak, over 25 just-in-time laboratories were established throughout the region, originating from such diverse sources as both the US Army and Navy, the European Mobile Laboratory cooperative, and Canadian, Chinese, Nigerian, Russian, Senegalese, and South African institutes. A challenge in interpreting data collected during the outbreak was that the samples were run on multiple instrument platforms with inconsistent access to proper assay controls. The rapidity of the response, austerity of the environment, limited supply chain, and even more limited cold chain supply management made procurement and testing of assay controls and validation of instrument platforms a challenge, though WHO made a bold effort at such coordination [31].

High-quality laboratory data are required to understand the safety and efficacy of experimental therapeutics. The experienced teams at MUWRP and IDI, both of which carry College of American Pathologists (CAP) accreditation, have been central to establishing the research capability at FPRRH. Working with principal investigators (PIs) from the IDI, MUWRP conducted a baseline survey of the hospital laboratory prior to JMEDICC initiation. The Fort Portal Regional Referral Hospital Laboratory (FPRRHL) was already participating in the Stepwise Laboratory Quality Improvement Process Towards Accreditation (SLIPTA), which provided an opportunity for the entire laboratory staff to work together toward achievable improvement in laboratory quality. Strengthening the existing laboratory quality management system (LQMS) included, but was not limited to, developing standard operating procedures (SOPs), equipment verifications, training staff on handling external quality assessment (EQA)/proficiency testing (PT) panels and utilizing the data to improve systems, and advancing staff training and staff records to meet the requirements of laboratory audits and reviews. On 21 May 2019, the hospital laboratory was accredited by the South African National Accreditation System (SANAS), a testament to the team’s hard work and quality management.

The US and Ugandan teams worked together to develop and initiate a GCLP training program because GCLP is critical to clinical research. To achieve the totality of this training is a gradual process requiring ongoing training and evaluation. JMEDICC has used various training tools, including staff trainings, mentorships, and competency assessments, to achieve a regulated environment that is supported by the entire team, many of whom had never before worked in clinical research. In order to maintain a high quality of work, the laboratory now utilizes a robust quality plan, which elaborates the requirements for sample testing, new equipment and/or assay verification, staff competency assessments, management of SOPs, and quality improvement processes. All project staff support this quality plan and are cross-trained on instruments and assays to permit maximum staffing flexibility in the outbreak response. Early in its work, the JMEDICC team recognized the dual importance of both high-consequence pathogen diagnostic capability and laboratory capacity for pharmacovigilance and clinical performance of care (hematology, chemistry, malariology). This additional focus moves the capability beyond the usual outbreak response laboratory structure in preparation for assessing safety and effectiveness of novel therapeutic products.

## IPC and biosafety

IPC principles may be introduced in a day but incorporating the principles into regular work practice requires regular and evolving training. JMEDICC IPC training is scheduled weekly, during which time rotating team members stand down from normal clinical operations and focus on training drills to support the management of VHF patients. The team benefits from direct institutional and field experiences by both US and Ugandan team members. The training has encompassed generic procedures, including the conduct of regular duties at a high-IPC posture, personal protective equipment (PPE) donning and doffing, spill management, and response to exposures or breaches in PPE.

At the time of recruitment, none of the project Ugandan laboratory staff had experience working with highly infectious pathogens like the Ebola virus. Having drilled in IPC procedures since July 2016, the team has now developed an understanding of IPC principles, and the training plan has similarly evolved. For more advanced IPC questions, the team works through processes and risk assessments in collaboration with external SMEs, developing a core capability for managing unexpected events. Jointly, the team has developed SOPs, diagrams, flow-charts, and checklists that standardize core procedures and reinforce IPC principles.

## Infrastructure and instrumentation

Infrastructure challenges were a significant impediment to clinical care and research in the West Africa Ebola virus outbreak. JMEDICC has invested in core infrastructure capabilities in an effort to mitigate similar risks (Table 1). All JMEDICC operations are supported with backup generators, permitting a reliable electrical source for operations, and a local area network has been established, permitting electronic communication within the research ward. Reliable electricity and established data management and communication channels enable the team to focus on study execution.

**Table 1:** Core capability considerations.

Challenge	Implications of Failure	Solution
**Reliable electricity**	Instrument damage and data loss.	Standby generators and inverter systems installed; all instrumentation on UPS systems.
**Waste management**	Buildup of highly infectious waste with no safe and efficient means of destruction, potentially resulting in spread of infectious material.	Renovated the FPRRH hospital incinerator, now capable of managing both outbreak and normal hospital waste flow. Established an isolated route between research unit and incinerator, allowing for safe management of highly infectious waste.
**Internet connectivity**	Complicates data capture, storage, and transfer; communication between partners; access to SOP sharing and EQA/PT panel data.	Established secure LAN server for project.
**Data management and communication**	Unreliable data transfer and unreliable clinical data to support clinical trial; slow transfer of critical patient data to the treating clinical team may impact patient outcome.	Established electronic data capture system, telephone lines between hot and cold zones, and shared network folders on secured LAN. Backup use of Nalgene Polyolefin paper, which can be written on and decontaminated with chlorine.

**Abbreviations:** EQA, external quality assessment; FPRRH, Fort Portal Regional Referral Hospital; LAN, local area network; PT, proficiency testing; SOP, standard operating procedures; UPS, uninterrupted power supply

Maintaining a reliable supply chain in a rural environment is essential for clinical trial execution. The JMEDICC logistics team manages cycle stock with both push and pull inventory delivery aspects, providing continuous and proactive support to both clinical and laboratory teams. This allows the staff to focus on their core competencies and improve their output.

Selection of instrumentation for JMEDICC required consideration of the project’s long-term goals: quality, safety, and mobility. The size and temperature requirements of supplies and instruments were considered, as were the proposed biocontainment requirements of each individual assay and piece of instrumentation. Unlike polymerase chain reaction (PCR), for which inactivated samples may be used, blood chemistry and hematology analysis must utilize unaltered, potentially infectious samples. It is therefore essential that instrumentation be housed in biosafety enclosures. Equally important were issues of supply chain and instrument maintenance contract availability that would enable maintenance of laboratory quality standards.

A total of 5 new instruments were selected for the JMEDICC project, allowing for blood chemistry analysis, hematology analysis, coagulation analysis, and diagnostics (Table 2). Rapid diagnostic testing for malaria was instituted and the Ugandan national algorithm for human immunodeficiency virus (HIV) testing was adopted. As the staff began utilizing selected instruments, modifications were made based on supply chain and verification issues. Subsequently, filovirus-specific assays were introduced. Although the primary diagnostic platform for JMEDICC is the rapid and simple BioFire FilmArray Warrior Panel, which has been cleared for filovirus diagnostics by the US FDA, the team is also being trained on PCR to enable quantitative viral load analysis. The team has passed competency assessments in nucleic acid extraction, standard curve dilution series, and blinded PCR sample analysis. This training will enable them to conduct high-quality research grade assays to detect pathogens of interest for which there are not FDA cleared or Emergency Use Authorization (EUA) assays available. The Cepheid GeneXpert instrument is also utilized onsite, which can be used to test for Ebola virus Zaire strain, among other pathogens.

**Table 2:** Instrument selection.

Instrument	Purpose	Benefits	Challenges
Piccolo Xpress	Blood chemistry analysis	• Small footprint • FDA-cleared assays • Electrolyte, liver and renal function in one test	• Runs single sample at a time • Length of testing time
Beckman Coulter DxH500 and DxH520	Hematology analysis	• 5-part differential • Small footprint • DxH520 is a closed system • DxH520 is FDA cleared	• DxH500 not FDA cleared
BFT II	Coagulation analysis (PT and aPTT)	• FDA cleared	• Somewhat complex sample preparation
BioFire FilmArray	Diagnostics	• Ruggedized and small footprint • Multiple targets in one assay • FDA cleared for Ebola, Sudan, and Marburg virus • Minimal processing and reduced chance of contamination compared to traditional PCR	• Qualitative result with no Ct/quantitative information • Results are partially restricted, so strain-specific data not always available • Runs single sample at a time
BinaxNOW	Malaria rapid diagnostic test	• Rapid test • FDA cleared	• Does not provide a definitive result for negative samples • Differentiation between a *Plasmodium falciparum* only infection and a mixed infection containing *P*. *falciparum*. and another malaria species is not possible
CFX96 PCR platform	High throughput diagnostics if necessary	• Higher throughput • Small, mobile platform • Provides Ct	• More complex sample manipulation (RNA extraction required) and higher chance of contamination

Abbreviations: aPTT, activated partial thromboplastin time; Ct, cycle threshold; FDA, Food and Drug Administration; PCR, polymerase chain reaction; PT, prothrombin time; RNA, ribonucleic acid

Clinical trials require instrumentation that can be verified for generating data that meet the rigor of FDA review, regardless of logistics issues such as reliability of supply chain and instrument size. For JMEDICC, almost all selected instruments and assays are US FDA cleared, simplifying the verification requirements for each instrument. One exception is the hematology instrument. A Beckman Coulter instrument, the DxH500, was selected for JMEDICC hematology analysis. This instrument is unique among hematology analyzers for its extremely small footprint coupled to the 5-part differential that it provides. Considering the impact of filovirus infection on peripheral blood cell populations like lymphocytes, the team felt that its use was justified in order to have a more thorough hematological assessment [32]. Recently, the next generation DxH520 instrument was successfully cleared by FDA, and the team is in the process of verifying that instrument.

GCLP guidelines require that an instrument be verified whenever it is moved. However, in the scenario of a filovirus clinical trial, it is possible that movement of equipment will be required regularly, and the laboratory team will not have the benefit of time to perform lengthy verifications. JMEDICC developed a mobile verification plan under which instruments are verified thoroughly at the FPRRH hub site and, upon movement, are reverified for accuracy and precision using stored clinical or control samples. This hybrid verification on site provides the team with confidence in the quality of their results without imposing an undesirable delay in their ability to use the instrumentation or a burden on staff to prolong time spent in the hot zone.

## Biocontainment operations

JMEDICC day-to-day operations take place at the FPRRH main laboratory and involve clinical laboratory testing of samples from patients enrolled in the sepsis study at the hospital. This laboratory is biosafety level (BSL) 2 and is not intended for testing of samples from patients known or suspected to be infected with highly infectious pathogens like filoviruses.

The JMEDICC research ward laboratory has biocontainment procedures approximating a cabinet BSL-4 operational stance. The space is not equipped with negative pressure, and there is no intention to culture or amplify pathogen; it serves strictly to support diagnosis and ongoing patient care at the research ward. Manipulation of infectious samples occurs in mobile, negative pressure, double high-efficiency particulate air (HEPA) filtered, glove box rapid containment kits (RCK) by Germfree. They provide an effective and flexible option for enhanced biosafety procedures. JMEDICC has affixed pressure monitors to these units, and the units receive hardwired power from the electrical system or a generator rather than relying on batteries, reducing the chance of battery failure. Moreover, as an outbreak may occur with patients presenting either to FPRRH or a remote site, all processes are designed with an eye toward translocation of operations to a mobile setting.

The laboratory biocontainment approach has focused on a no less than “double protection” principle. All processes and procedures are designed to have at least 2 levels of protection for personnel and the environment. Examples of typical personnel protection include practiced IPC procedures (such as enforced buddy system and monitoring), appropriate PPE using the MAXAir powered air purifying respirator (PAPR), and sealed sample tubes. Protection during specimen handling is enhanced with closed secondary containers and employment of the RCK. Waste management practices and use of the RCK in a controlled workspace afford environmental protection.

The team has practiced with an aerosolized hydrogen peroxide (aHP) mist for sanitizing spaces after working with highly infectious samples, including instruments, pipets, cabinets, and containment hoods. The mist can be dispensed with a handheld sprayer while personnel are in full PPE, and then the hydrogen peroxide can be aerosolized automatically by the dispenser to sanitize the entire space. Although chlorine decontamination will be the primary unit decontamination standard, the aHP will be utilized in both stationary and mobile response operations as a redundant safety measure.

## Biosecurity

JMEDICC is committed to ensuring an appropriately secure work setting, safe sample storage, and inventory tracking. The JMEDICC research ward is designed to accommodate high-security storage requirements, which includes biometric access to the laboratory and other secure spaces. Pin code access is used when the unit is active, and PPE prevent the use of biometrics. Refrigerators and freezers are secured with locks and temperature monitors for security of product and samples. An unobtrusive perimeter security is maintained with fencing and guards, and entry and exit of personnel are documented by the security guards. Inventory of both supplies and samples is carefully tracked using an electronic laboratory management information system, all to ensure the integrity and security of samples.

## Sample storage and shipment

A continual obstacle to advancing our understanding of rare, high-consequence pathogen infection is our inability to study infected patients or clinical specimens. JMEDICC has established biosecurity procedures and sample storage resources to maximize our ability to learn from residual clinical samples. Upon concurrence of necessary authorities, both untouched frozen and inactivated samples from infected, consenting subjects will be stored temporarily at the JMEDICC site and then transported to biosafety level 4 laboratories in the US. Primary research aims include pharmacokinetic analysis of subjects treated with investigational products in order to determine whether the product achieves desired levels in sick patients, viral sequencing to examine strain variants as well as virus evolution in response to treatment, and investigational endpoints, including immunological analyses of the host response to infection. The US Centers for Disease Control (CDC) import permits are in place to enable receipt of samples in the US, and members of the staff are International Air Transport Association (IATA) trained, ensuring that samples will be packaged appropriately for shipment.

## Stakeholder engagement

Stakeholder engagement has been a central part of every aspect of JMEDICC, but it has a unique role in laboratory efforts. The initial pathogens of interest for JMEDICC clinical studies are filoviruses. These high-consequence pathogens garner interest from numerous domestic and international stakeholders.

Uganda has a well-established outbreak preparedness and response approach led by the National Task Force (NTF), which integrates teams from the MoH, the local district health offices, and international stakeholders, such as the US CDC, WHO, and Médecins Sans Frontières (MSF) [33, 34]. The Uganda Virus Research Institute (UVRI) is the only laboratory approved by the Ugandan government to test samples from patients identified as suspect (or “under investigation”) VHF cases. It is incumbent upon new entities such as JMEDICC, therefore, to integrate into the existing structure without negatively impacting patient diagnosis and care. MUWRP, IDI, and other partners attend NTF meetings to ensure that JMEDICC conforms to the national outbreak response approach.

JMEDICC continually undertakes social mobilization and seeks community advice both for sepsis research and program improvement. To ensure that the team was well informed about the national outbreak preparedness procedures in Uganda, JMEDICC executed a joint exercise with UVRI and the NTF in December 2017. During this exercise, a mock VHF patient presented at the hospital and was moved to the research ward, and a sample was collected for diagnostic testing. In close partnership with the hospital and district health office, the sample was sent to UVRI for accessioning and testing; subsequently, the result was reported back to the NTF, hospital, district health office, and ultimately to JMEDICC staff. This exercise enabled the team to apply written guidance to a real-life scenario and to identify critical aspects of the communication loop. Exercises such as these are a core component of building relationships and procedures for operating in a filovirus outbreak environment.

## Mobile capability

Although the FPRRH was selected as the hub base for JMEDICC activities because of its location in a region that has experienced endemic filovirus disease, not all filovirus sentinel cases in Uganda will present at the FPRRH. The hub base has been useful in providing familiarity to high-consequence pathogen case management, and in-country personnel have worked with local authorities in response to alert cases, including a Crimean–Congo hemorrhagic fever (CCHF) case, which was managed successfully at the JMEDICC research ward. The ultimate goal of JMEDICC, however, is to be able to field the capability to where the country is experiencing an outbreak. In October 2017, JMEDICC US personnel provided early technical assistance during the country’s Marburg disease outbreak in Kween and Kapchorwa, in parallel with IDI and MUWRP, which supported response efforts in their usual roles with the NTF. Lessons from that experience and the program’s continued work as part of the broad coalition of domestic and international partners in Uganda keep attention on the need to further develop mobile capability for clinical trial work.

Determining the scope and capabilities of a mobile laboratory requires compromise and balance. Laboratory assay availability can drive the patient management approach of the clinical team, so constant communication between laboratory and clinical teams is essential to ensure that the resources of the laboratory team meet the needs of clinical care. However, the laboratory team also needs to ensure that they do not overextend in an outbreak setting. Factors contributing to mobile capabilities include supply chain, as assay controls for most instruments require cold chain and often −20°C or −80°C storage conditions; personnel coverage, as increasing the number and complexity of assays requires that an increased number of staff be available; and clean, reliable power. Current mobile plans focus on diagnostics and blood chemistry analysis, which are critical for pathogen identification and safety assessments of administered therapeutics. Additional resources would also enable deployment of hematology and coagulation analyzers, which instruments have been selected specifically for their small footprint to enable mobility. The final laboratory test menu needs to be assessed in the context of the training and size of the laboratory team, considering the stressful conditions associated with operating in a filovirus environment at a high-IPC posture with heat-intolerant PPE. JMEDICC is preparing for these challenges in concert with Ugandan and international partners, whose assistance with logistical challenges would expedite deployment of mobile lab resources.

## Current project status: 2018/2019 Ebola outbreak in the DRC

Since August 2018, an Ebola virus outbreak has been spreading through the eastern DRC, resulting in 3,288 cases and 2,192 deaths as of 13 November 2019. Three Ebola virus–infected individuals were identified in Uganda in June 2019, resulting in concern that this would mark the international spread of the outbreak. However, the 3 cases were contained, and no local transmission occurred in Uganda.

JMEDICC has approval to operate only in Uganda and cannot at this time cross into DRC to support outbreak response there. Within Uganda, however, JMEDICC staff participate in the NTF of Uganda and work with the District Task Force for Kabarole District, which leads the local response in the district. In collaboration with the FPRRH, the project has helped establish a suspect patient ward at the hospital and trained local HCWs in IPC as well as patient management. In all situations, the team follows the lead of the MoH and WHO. The Ugandan MoH has requested that the JMEDICC site be made available as a HCWs treatment unit, and the team is standing by to support that request. In addition, JMEDICC members participate in calls and regular communication with WHO to ensure that project therapeutics efforts align with WHO guidance on specific pathogens. In terms of laboratory-specific support, the JMEDICC laboratory team has had numerous discussions with the US CDC and UVRI about engagement in subnational testing and collaboration with UVRI on diagnostic testing, and the team looks forward to assisting should the MoH desire. At the time of writing, JMEDICC has an approved MEURI protocol for remdesivir in place and is working with external partners to collaborate on a MEURI protocol for mAb114. The team is also in discussions about collaborating on protocols to make REGN-EB3 available.

## Conclusions and future directions

Today, the JMEDICC-empowered MUWRP–IDI team is a sustainable and agile entity capable of rapidly and effectively responding to outbreaks in Uganda. Efforts by the team will support collection of data that can aid in licensure of promising medical countermeasures targeting high-consequence pathogens like filoviruses. Although the JMEDICC team is equipped to respond to filovirus outbreaks, the potential for research and response activities is certainly not restricted to such an end. The expertise of the team in Fort Portal has numerous applications, including conduct of Phase I vaccine or therapeutics trials in endemic populations, serosurveillance studies, testing of diagnostic assays relevant to pathogens in the region (including CCHF and Rift Valley fever virus), and more.

**Box 1. Perspectives from JMEDICC Staff**

1. **As a new laboratory technician on the JMEDICC staff who has never worked in clinical research before, what are some of the challenges you have faced?**
  - **Training on new assays, procedures and protocols required for the research**. In a clinical research study setting, trainings like GCLP and Collaborative Institutional Training Initiative (CITI) are more critical than in a research lab setting.
  - **Stringent documentation procedures**. Attention to detail is very crucial in a clinical research setting. All information has to be captured and owned by the personnel in real time. Documentation of personnel training, competency assessment, and immunization must be captured. SOPs must be in place for all platforms and processes. In a regular laboratory setting, there is less focus on documentation.
  - **Assay and equipment validation and verification**. In a regular research lab setting, validation and verification of processes is less stringent; in a clinical lab setting, all methods have to be verified to ensure that results from the equipment and assays are reproducible, repeatable, and comparable and that the methods will achieve results in accordance with the manufacturer’s specifications. This ensures that credible results are obtained for patient management and data obtained can be used to evaluate a clinical trial.
  - **More stringent quality control measures**. In a regular high-volume laboratory setting, it is more likely that the LQMS will be more flexible to accommodate the large volume of testing. In a clinical research laboratory setting, procedures are more streamlined and have to be adhered to with some level of firmness. For example, process controls have to be performed at a specified interval, participation in EQA for external monitoring of performance on all platforms has to be observed, and routine temperature monitoring, continuous quality improvement through implementation of quality improvement projects and routine equipment service and monitoring have to be performed.
  - **Inventory management**. In a clinical lab setting, measures have to be put in place to ensure that there is proper stock management and inventory systems. A mechanism of ordering, receiving, checking, and utilization of supplies must be put in place, and suppliers informed in advance for proper planning. These methods have to continuously be observed and checked to ensure they are working. In a regular lab setting, methods are not rigorously observed.
  - **Data management**. In a clinical research setting, data have to be managed in a way that protects participant identity and have to undergo various levels of quality control to ensure that the right entries are captured. Data management in a regular lab setting is not as rigorous.
2. **As a laboratory technician who has not previously worked in an outbreak setting, what are the challenges you face operating in an outbreak setting as opposed to a normal hospital setting?**
  - **Environmental changes**. Changes in working environment, like temperature, need acclimatization, which may take time for the team. This may result in heat stress and dehydration from using heavy PPE if the environmental temperatures of the place are higher.
  - **Organization of the VHF treatment unit in times when there might be PPE failure**. VHF treatment units tend to be organized in such a way that the doffing lane may not be located near the laboratory or clinical area as is the case in the IPC drills at FPRRH. Personnel who have had a PPE failure (specifically PAPR) may not be able to handle walking long distances to doff.
  - **Long working hours in PPE, given the staff hired and type of procedures performed.** Some procedures in the laboratory take time and, given that the staff are working in shifts, there may be a challenge of long working hours.
  - **Variations in training on protocol and PPE with different implementing partners.** Working with other partners in the VHF treatment unit may require the team to adjust the PPE posture, which will require time to get acclimatized. There may be differences in procedures like spill management and waste management, which may cause confusion in the team.
3. **As a part of JMEDICC laboratory leadership, what are some of the most difficult challenges in establishment of the project?**
  - Achieving GCLP and the critical level of laboratory quality, consistency, and accountability, is more complex in an outbreak setting when the team is working with highly infectious pathogens. The balance between strict GCLP adherence and biosafety is a constant consideration as every moment a team member is in the hot zone is a moment when they may be exposed to pathogen. The more tired team members are, the higher the likelihood of a safety breach, which means that as leaders, we must balance the requirements of good laboratory practice with the safety of the staff to ensure that they are not doing unnecessary tasks on the hot side.

## Supporting information

S1 Data JMEDICC Consortium Members. JMEDICC Consortium encompasses personnel who are actively engaged in the project development and execution or were critical to project inception and establishment. These personnel have contributed substantively to the overall development of the JMEDICC capability and are considered authors on this manuscript. JMEDICC, Joint Mobile Emerging Disease Clinical Capability
(DOCX)
