# Supplementary material for: The Joint Mobile Emerging Disease Clinical Capability (JMEDICC) laboratory approach: Capabilities for high-consequence pathogen clinical research
Source: PLoS Negl Trop Dis. 2019 Dec 19;13(12):e0007787. doi: 10.1371/journal.pntd.0007787 (PMC6922336; doi:10.1371/journal.pntd.0007787)
Supplement: S1 Data — JMEDICC Consortium encompasses personnel who are actively engaged in the project development and execution or were critical to project inception and establishment. These personnel have contributed substantively to the overall development of the JMEDICC capability and are considered authors on this manuscript. JMEDICC, Joint Mobile Emerging Disease Clinical Capability (DOCX) [file pntd.0007787.s001.docx]

**Supplemental Data 1. JMEDICC Consortium Members**

Rodgers Ayebare, MD, Infectious Diseases Institute Makerere University, Kampala, Uganda

Andrew Baguma, Infectious Diseases Institute Makerere University, Kampala, Uganda

Nahid Bhadelia, MD, Medical Director, Special Pathogens Unit, Boston University School of Medicine, Boston, MA, USA

David Brett-Major, MD, Navigating Health Risks, LLC, Bethesda, MD, USA, 20817

George Christopher, MD, formerly of JPEO-Medical Countermeasures Systems, Fort Belvoir, VA, USA

Trevor A. Crowell, MD, PhD, Henry M. Jackson Foundation for the Advancement of Military Medicine, Bethesda, MD, USA

Commander Ben Espinosa, MD, Biological Defense Research Directorate at Naval Medical Research Center, Frederick, MD, USA

Anne Fox, MD, Navy Medical Research Unit No.3 Ghana Detachment

William Hulsey, Henry M. Jackson Foundation, Bethesda, MD, USA

Fatim Jallow, PhD, QUALABS Consulting, Inc. Hartford, CT, USA

Francis Kakooza, Infectious Diseases Institute Makerere University, Kampala, Uganda

Francis Kiweewa, MD, Makerere University Walter Reed Project, Plot 42 Nakasero Road, Kampala, Uganda

Mike Kozlowski, JPEO-Medical Countermeasures Systems, Fort Belvoir, VA, USA

James Lackemeyer, US Army Medical Research Institute of Infectious Diseases, Fort Detrick, MD, USA

James Lawler, MD, MPH, University of Nebraska Medical Center, Omaha, NE, USA

Captain Suzanne Mate, PhD, Walter Reed Army Institute of Research, Bethesda, MD, USA

Raymond Mayanja, Makerere University Walter Reed Project, Plot 42 Nakasero Road, Kampala, Uganda

Monica Millard, Walter Reed Army Institute of Research Program Director, Kampala, Uganda

Ezra Musingye, Makerere University Walter Reed Project, Plot 42 Nakasero Road, Kampala, Uganda

Susan Nabukenya, Makerere University Walter Reed Project, Plot 42 Nakasero Road, Kampala, Uganda

Joaniter Nankabirwa, MD, formerly of Makerere University Walter Reed Project, Plot 42 Nakasero Road, Kampala, Uganda

Arthur Natwijuka, Makerere University Walter Reed Project, Plot 42 Nakasero Road, Kampala, Uganda

Stephen Okello, MD, Makerere University Walter Reed Project, Plot 42 Nakasero Road, Kampala, Uganda

Rachel Overman, JPEO-Medical Countermeasures Systems, Fort Detrick, MD, USA

Marilyn Powers, Henry M. Jackson Foundation, Bethesda, MD, USA

Allan Tindikahwa, Makerere University Walter Reed Project, Plot 42 Nakasero Road, Kampala, Uganda

Peter Waitt, MD, Infectious Diseases Institute Makerere University, Kampala, Uganda
